# Ubiquitin C-Terminal Hydrolase L5 (UCHL5) Accelerates the Growth of Endometrial Cancer via Activating the Wnt/β-Catenin Signaling Pathway

**DOI:** 10.3389/fonc.2020.00865

**Published:** 2020-06-11

**Authors:** Da Liu, Zixuan Song, Xiaoying Wang, Ling Ouyang

**Affiliations:** Department of Obstetrics and Gynecology, Shengjing Hospital of China Medical University, Shenyang, China

**Keywords:** endometrial cancer, UCHL5, lentivirus vectors, Wnt/β-catenin pathway, XAV939

## Abstract

Endometrial cancer (EC) is the most prevalent gynecological malignancy with high mortality. Chemotherapy plays a pivotal role both in an adjuvant setting and in exclusive treatment. However, current pharmacotherapies are limited and not ideal for improving the overall survival of EC patients. Thus, identification of the underlying molecular mechanisms responsible for initiation and progression of EC is imperative for developing novel therapeutic strategies. Ubiquitin C-terminal hydrolase L5 (UCHL5) has been found to aggravate tumor growth and metastasis in several different types of tumor models such as esophageal squamous cell carcinoma, hepatocellular carcinoma, and epithelial ovarian cancer. However, whether UCHL5 influences the growth of EC has not been elucidated. To expose the role of UCHL5 on EC, bioinformatics analysis was conducted, and it hinted that UCHL5 was overexpressed in EC tissues and associated with lower overall survival. Consistently, the overexpression of UCHL5 in EC tissues and cell lines was further confirmed by western blot (WB) and polymerase chain reaction (PCR) compared with non-tumor control. Lentivirus vectors carrying UCHL5 shRNA or CD sequences were used to reduce or overexpress the UCHL5 gene, respectively. Cell proliferation and cycle were facilitated, and cell apoptosis was decreased when the UCHL5 gene was overexpressed in EC cell lines. These results were opposite in UCHL5 knockdown EC cells. Additionally, the expression of β-catenin is positively related to UCHL5 levels and the tumorigenic effects of UCHL5 overexpression were reversed by the Wnt/β-catenin pathway inhibitor XAV939. Thus, Wnt/β-catenin pathway activation may be a partial mechanism responsible for the promoting effects of UCHL5 on EC growth. In conclusion, UCHL5 accelerated the growth of EC via the Wnt/β-catenin pathway and was expected to be an attractive target for EC treatment.

## Introduction

Endometrial cancer (EC) is the most prevalent invasive gynecological malignancy in the western world and the fourth common cancer in women worldwide, with over 280,000 new cases worldwide in 2017 (1). The morbidity is still climbing due to the prevalence of global obesity, the coming of aging society, environmental degradation, etc. (2). Although most patients are diagnosed early and accepted comprehensive treatments, the number of estimated deaths caused by EC was almost 36,000 annually, accounting for 1.8% of all cancer deaths (3). More notably, if without follow-up adjuvant therapies such as chemotherapy or radiotherapy, 10–15% surgical patients will relapse (4). These recurrent cases have a poor prognosis with a 5-year survival rate less than 15% because of the limited treatment options and resistance to current medications (4, 5). Therefore, deeper molecular mechanisms and targeted drugs deserve further exploration.

The ubiquitin–proteasome system (UPS) is known as a specific and selective pathway for most protein degradation in all eukaryotic cells (6). Ubiquitin (Ub), a small 76-residue protein, marks these proteins who are destined for degradation in the form of polymeric chains so that they were accurately recognized and degraded by the proteasome (7). The process of ubiquitination can be reversibly regulated through lengthening or cleaving the poly-Ub chains mediated by ubiquitinating and deubiquitinating enzymes (DUBs), respectively (7, 8). Previous investigations have demonstrated the UPS involved in various tumor-promoting processes, including DNA repair, apoptosis, cell cycle, and oncogenic signaling (9). Not surprisingly, the ubiquitination pathway has been considered as a fascinating target for anti-cancer drug research, especially after two proteasome inhibitor drugs (bortezomib and carfilzomib) are successfully approved for treatment of relapsed or refractory multiple myelomas (10). However, the actions of each component in the ubiquitination pathway on some solid tumors, especially EC, remain unclear.

According to the homology and the functional mechanism, DUBs are mainly segmented into five categories: Ub C-terminal hydrolase (UCH), Ub specific protease (USP), ovarian tumor protease (OTU), Josephin/Machado–Joseph disease protease (MJD), and JAB1/MPN/MOV34 metalloenzyme (JAMM) (11). Among 80 cloned DUBs in the human genome, nearly half of them turned out to be associated with the development of various types of cancer, previously (12). Ubiquitin C-terminal hydrolase L5 (UCHL5)/Uch37, a cysteine protease from the family of ubiquitin C-terminal hydrolases (UCHs), can remove Ub from the distal part of the poly-Ub chains and rescues poorly ubiquitinated proteins from proteolysis (13). Disabling the UCHL5 gene in mice results in embryonic death, indicating that UCHL5 function is very crucial (14). Overexpression of UHCL5 has been reported in several solid tumors and correlates with the poor survival and increased risk of cancer recurrence (15). Additionally, bAP15, a depressor of the 19S proteasome DUBs (UCHL5 and USP14), exhibits significant inhibition of cell growth and invasion in several human cancers (16). In conclusion, these researches indicated that UCHL5 plays a momentous role in the development of tumors.

The Wnt/β-catenin pathway controls various cellular processes of cancer biology, such as cell proliferation, differentiation, and maintenance of pluripotency (17). The function of the Wnt/β-catenin pathway on EC growth had been well-established (18). β-Catenin maintains epithelial cell integrity, an important barrier for blocking tumor metastasis (19). Additionally, hypophosphorylated β-catenin transfers into the nucleus and binds to transcription factors, leading to transcriptional activation of specific target genes, including Cyclin D1, C-MYC, and MMP-7, all of which contribute to cell survival, cell cycle, uncontrolled proliferation, and distant metastasis (20).

However, the link between UCHL5 and the Wnt/β-catenin pathway and the function of UCHL5 on EC growth have not yet been completely elucidated so far. In this study, we mainly want to solve the following questions: (1) Whether the expression of UCHL5 is significantly different between EC and normal endometrium; (2) How about the effects of UCHL5 on the growth and survival of EC; and (3) Whether the Wnt/β-catenin pathway can partly explain the effects of UCHL5 on EC. Our study may contribute to unravel the mechanisms underlying the occurrence and progression of EC and provide new therapeutic targets.

## Methods and Materials

### EC Cancer and Adjacent Tissue Collection

All the EC and adjacent non-neoplastic endometrial tissues were obtained from hysterectomy patients diagnosed as EC at the Department of Obstetrics and Gynecology, Shengjing Hospital affiliated with China Medical University, from 2016 to 2017. The EC tissues (*n* = 10) and tumor adjacent tissues (*n* =10) were separated from excised uteruses and then rapidly frozen with liquid nitrogen and stored at −80°C. All patients voluntarily signed an informed consent to donate their excised organ. Additionally, all patients had not received any chemotherapy or radiotherapy before their surgery. Besides, all tissue specimens were reviewed by a pathologist to confirm the diagnosis. This study was approved by the Ethics Committee of the Shengjing Hospital affiliated with China Medical University.

### Cell Lines and Cell Culture

Human endometrial cancer cell AN3-CA (ATCCHTB-111) was purchased from the American Type Culture Collection (ATCC). Other endometrial cancer cells HCE-1-A (BNCC338711), HCE-1-B (BNCC100172), Ishikawa (BNCC338359), and human endometrial epithelial cell hEEC (BNCC341726) were purchased from BeNa Culture Collection (BNCC). Each cell was cultured in a basic medium recommended by their user manual, supplemented with 10% fetal bovine serum (FBS) (GIBCO BRL, BRA, USA) and 100 U/mL penicillin and 100 μg/mL streptomycin (Beyotime Biotechnology, SH, China). Cell culture was carried out at 37°C, 5% CO_2_, and 95% humidity, and cell passage was digested by 0.25% (w/v) trypsin containing 0.53 mM EDTA (20).

### Lentiviral Vector Construction and Cell Transfection

Lentiviral-mediated shRNA interference technology was used to downregulate the UCHL5 gene in HCE-1-A cells. Three pairs of shRNA oligonucleotides were designed by GeneChem Company (Gene, SH, China) based on the sequence of the UCHL5 gene (NM_015984.5) and listed in Table 1. Each nucleotide sequence was inserted into the lentivirus core plasmid pLKO.1 (Addgene) after being digested by Agel I/EcoR I at 37°C. For lentiviral production, the recombinant plasmid described above and two packaging plasmids (psPAX2 and pMD2G) were co-transfected into HEK293T cells (ATCC) using Lipofectamine 2000 (Invitrogen) according to the manufacturer's instruction described previously (21). Forty-eight to seventy-two hours later, the virus supernatant was harvested and concentrated by ultracentrifugation (4,000 g at 4°C) for 10 min.

**Table 1:** Sequences of shUCHL5.

**shUCHL5**	**Sequence (5**′**-3**′**)**
shUCHL5-F1	CCGGTCCGAGCTCATTAAAGGATTCTCGAGAATCCTTTAA TGAGCTCGGTTTTTG
shUCHL5-R1	AATTCAAAAACCGAGCTCATTAAAGGATTCTCGAGAATCC TTTAATGAGCTCGGA
shUCHL5-F2	CCGGTCCGATTGATTTAGGTGCATCTCGAGATGCACCTAA ATCAATCGGTTTTTG
shUCHL5-R2	AATTCAAAAACCGATTGATTTAGGTGCATCTCGAGATGC ACCTAAATCAATCGGA
shUCHL5-F3	CCGGTGCAGAAGATAGCAGAGTTACTCGAGTAACTCTGC TATCTTCTGCTTTTTG
shUCHL5-R3	AATTCAAAAAGCAGAAGATAGCAGAGTTACTCGAGTA ACTCTGCTATCTTCTGCA

We used the lentiviral vector system to establish a human endometrial cancer AN3-CA cell stably overexpressing UCHL5 (oeUCHL5). The complete coding sequences (CDs) of UCHL5 were amplified by the primers listed in Table 2 and inserted into the core plasmid pLVX-Puro (Clontech, Laboratories, Inc.) after being digested by EcoR I/BamH I at 37°C. The recombinant lentiviral vector encoding the UCHL5 gene was produced according to the protocol described above.

**Table 2:** Sequences of UCHL5 CDs region.

**oeUCHL5**	**Sequence (5**′**-3**′**)**
UCHL5-F	CGGAATTCATGACGGGCAATGCCG
UCHL5-R	CGGGATCCTCATTTGGTTTCCTGAGCTTTC

For cell infection, cells were planted at a density of 50,000 cells per well in six-well-plates and then, respectively, transduced with purified lentiviruses at a multiplicity of infection of 40 (22). UCHL5 knockdown HCE-1-A cells were established by lentiviral infection containing UCHL5 shRNA sequences (shUCHL5) and UCHL5 overexpression AN3-CA cells were established by lentiviral infection containing UCHL5 CDs. Twenty-four hours after transduction, the medium was replaced and cells were incubated in a CO_2_ incubator at 37°C for later experiments.

### Bioinformatics Analysis

Public data of UCLH5 gene expression in EC patients were acquired from the Cancer Genome Atlas Project (TCGA; http://cancergenome.nih.gov/). The differences of UCLH5 gene mRNA expression levels were analyzed between 548 EC and 35 non-tumor specimens. The overall survival of EC patients with high and low UCLH5 expression was also obtained from the TCGA database. We used the gene set enrichment analysis (GSEA) approach to analyze the effects of UCLH5 differential gene expression on biological annotation and pathways. Enrichment results should satisfy *P* < 0.05 with FDR *q* < 0.25 in the present study.

### Cell Viability Assay

The cells were planted in 96-well-plates, and the final cell density was adjusted to 3 × 10^3^/well with fresh medium for 12, 24, 48, and 72 h after lentiviral infection. Cell Counting Kit-8 (Beyotime Biotechnology, SH, China) was used to analyze the cell viability according to the previous description (20).

### Cell Apoptosis

For cell apoptosis, UCHL5 knockdown HCE-1-A cells and UCHL5 overexpression AN3-CA cells treated with or without XAV939 (HY-15147, MedChemExpress, New Jersey, USA), a Wnt/β-catenin pathway inhibitor, were collected and then incubated with Annexin V-FITC and Propidium Iodide (PI) in a cassette according to the introduction of Apoptosis Detection Kit (C1062, Beyotime) (23). These labeled cells were directly delivered to a flow cytometer (Becton–Dickinson, Franklin Lakes, NJ, USA) with CellQuest software (BD Biosciences) to analyze the proportion of apoptotic cells without washing.

### Cell Cycle Analysis

For cell cycle analysis, pretreated cells were harvested and fixed by ice-cold 70% ethanol for 2 h at 4°C. Then, cells were incubated by RNase and PI reagent in the darkness according to the Cell Cycle Analysis Kit (C1052, Beyotime) protocol (20). After a 30-min incubation, cell cycle was analyzed by a flow cytometer (NovoCyte, Aceabio, San Diego, CA).

### Western Blot (WB)

After being washed with phosphate-buffered saline (PBS), patient tissue specimens or pretreated cells were lysed in RIPA lysis buffer (Beyotime) containing 1% protease inhibitor (Beyotime). All samples were centrifuged at 16,000 × g for 20 min at 4°C, and then the supernatant was used for protein quantification by BCA protein assay kit (Beyotime). Then, proteins that underwent thermal denaturation at 98°C for 10 min were separated by SDS-PAGE electrophoresis and transferred to polyvinylidene difluoride (PVDF) membranes (Millipore). Subsequently, 5% non-fat milk in Tris-buffered saline containing 0.1% Tween 20 (TBST) was used to block the membranes for 1 h on a rolling device at room temperature. Removing blocking solution, corresponding primary antibodies were added and incubated overnight at 4°C. Then, these membranes were incubated with horseradish peroxidase (HRP)–conjugated secondary antibodies for another 2 h at room temperature after being washed six times with TBST buffer. The information of antibodies was below: Survivin (Ab469, Abcam, 1:5,000 dilution), β-catenin (Ab32572, Abcam, 1:5,000 dilution), UCHL5 (11527-1-AP, Proteintech, 1:500 dilution), CyclinD1 (Ab16663, Abcam, 1:200 dilution), Cleaved-Caspase3 (Ab4051, Abcam, 1:200), C-myc (Ab185656, Abcam, 1:2,000 dilution), Goat anti-mouse (A0216, Beyotime, 1:1,000 dilution), and Goat anti-rabbit (A0208, Beyotime, 1:1,000 dilution) HRP-labeled secondary antibody. GAPDH (60004-1-1G, Proteintech, 1:5,000 dilution) was used as an internal control in this study. The relative intensity of proteins was analyzed with Image Lab software (Bio-Rad) after interacting with enhanced chemiluminescence (ECL) chromogenic substrate (Beyotime).

### Quantitative Real-Time PCR (qRT-PCR)

For analysis of relative mRNA level, total RNA extraction from cells or tumors was performed using TRIzol reagent (Invitrogen, Carlsbad, CA, USA), and then reverse-transcribed into cDNA using the M-MLV First-Strand cDNA Synthesis Kit (Invitrogen) (23). The mRNA levels of the UCHL5 gene were quantified by the qRT-PCR method with Power SYBR Green PCR Master Mix (Invitrogen). The primers are listed in Table 3. An Applied Biosystems 7500 Fast Dx Real-Time PCR instrument (Thermo Fisher, USA) was used in the study. GAPDH was taken as an internal control and the results were analyzed using the 2^−ΔΔCt^ method.

**Table 3:** Primer of RT-PCR.

**Genes**	**Sequence (5**′**-3**′**)**
UCHL5-F	ATGAAAGGCTTGGCACTG
UCHL5-R	CTCCCATTAACAGGAACATAAC
GAPDH-F	GGATTGTCTGGCAGTAGCC
GAPDH-R	ATTGTGAAAGGCAGGGAG

### Animal Model

HCE-1-A cells with or without shUCHL5 lentiviral infection were separated by 0.25% trypsin, and the concentration of the cell suspension was modulated to 5 × 10^6^ cells/100 μL, then 100 μL cell suspension was injected into the left lower lateral subcutaneous tissue of the nude mice (20). Mice were considered tumor bearing when tumors became palpable at 12 days after the first injection and included into this study. Tumor growth was measured with a caliper using the formula *V* = *W*^2^*L*π/6, where *V* represents tumor volume, *W* means short diameter, and *L* is the mean long diameter. The volume of the xenografts was measured and recorded every 3 days, and the mice were euthanized for 33 days with the tumors separated and weighed.

### Statistical Analysis

Data are presented as means ± SEM and statistically analyzed by GraphPad Prism 4.0 software (GraphPad Software). The numbers of repetitions and groups about each experimental section are listed in the figure legends. Statistical analysis was based on Student's *t*-test or a one-way ANOVA with Tukey's *post-hoc* test according to fact conditions. For all tests, a *P*-value of < 0.05 was considered significant.

## Results

### The Expression of UCHL5 Was Significantly Increased in EC Tissues

**Figure 1:**
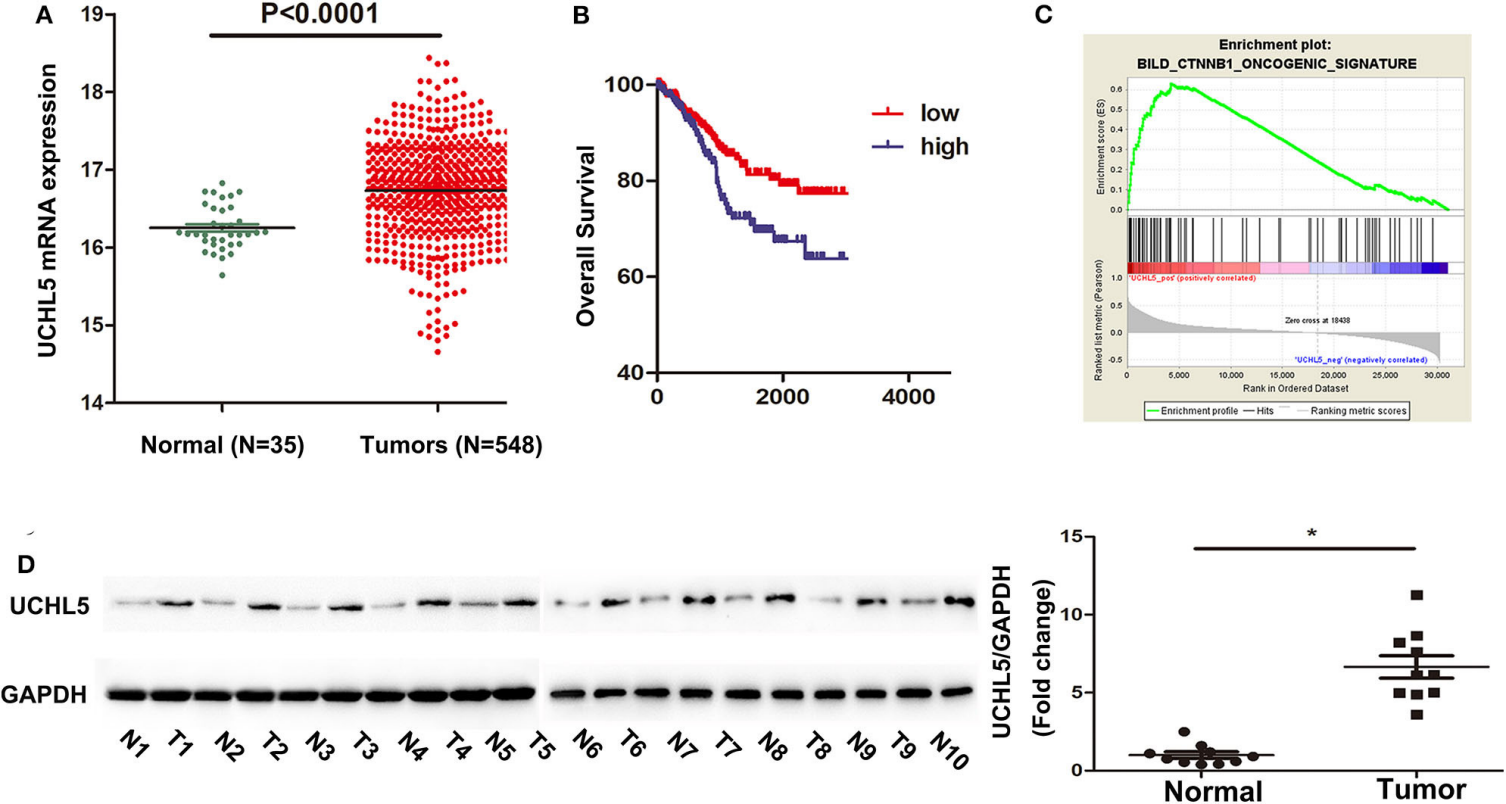
The expression of UCHL5 was significantly increased in EC tissues and negatively correlated with overall survival. **(A)** The relative levels of UCHL5 mRNA expression between EC tissues (*N* = 548) and non-tumor specimens (*N* = 35) were obtained from the TCGA database. **(B)** Overall survival (OS) was analyzed between patients with high UCHL5 and low UCHL5 expression in the TCGA website. **(C)** Representative image of gene set enrichment plots. **(D)** Western blot detected the expression of UCHL5 protein between 10 EC tissues (abbreviated to “T”) and matched adjacent normal tissues (abbreviated to “*N*”) (*N* = 10). *P < 0.05 vs. matched adjacent normal tissues.

In order to find out the levels of UCHL5 expression in EC samples, we first analyzed UCHL5 mRNA expression by comparing 548 EC tissues and 35 normal endometrial tissues from the TCGA database. As shown in Figure 1A, the relative UCHL5 expression level significantly increased in EC tissues compared with non-tumor specimens. Overall survival (OS) of patients with high UCHL5 mRNA levels and low UCHL5 levels was compared using the TCGA database. The results suggested that EC patients with low UCHL5 mRNA levels had markedly higher OS than those with high UCHL5 mRNA levels (Figure 1B). To analyze the potential biological functions of UCHL5, gene set enrichment analysis (GSEA) was conducted as previously reported (24). Among them, GSEA enrichment plots showed that enrichment of CTNNB1 (the coding gene for β-catenin) was associated with UCHL5 gene (Figure 1C). Consistent with the bioinformatics results, UCHL5 protein detected by WB was overexpressed in EC tissues compared with matched adjacent normal tissues (Figure 1D).

### UCHL5 Richly Expressed in Endometrial Cancer Cell Lines

**Figure 2:**
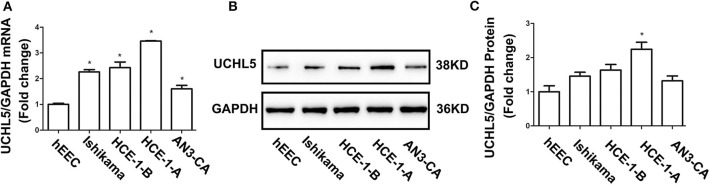
UCHL5 expressed in different endometrial cancer cell lines. **(A)** Relative mRNA levels of UCHL5 measured by qRT-PCR in human endometrial epithelial cell hEEC and four endometrial cancer cells (Ishikawa, HCE-1-B, HCE-1-A, AN3-CA) (*N* = 3). *P < 0.05 vs. hEEC. **(B)** Representative bands of UCHL5 and GAPDH in tumors were evaluated by western blot. **(C)** Statistical diagram of protein levels of UCHL5 (*N* = 3 times). *P < 0.05 vs. hEEC.

Based on the aforementioned results, four human EC cell lines (HCE-1-A, HCE-1-B, AN3-CA, Ishikama) and one human endometrial epithelial cell hEEC were selected to test the UCHL5 expression by qRT-PCR and WB. As shown in Figure 2A, the levels of UCHL5 mRNA were higher in four EC cell lines than those in hEEC cells. Among four human EC cell lines, its expression was highest in HCE-1-A cells and lowest in AN3-CA cells. Consistent with RT-PCR results, UCHL5 proteins were higher in EC cell lines to various extents than those in human endometrial epithelial cells (Figures 2B,C). The cell lines with higher expression of UCHL5 mRNA also presented higher expression of UCHL5 protein, suggesting that UCHL5 mRNA stably existed in EC cells and was translated into protein.

### UCHL5 Knockdown Inhibited the Growth of Endometrial Cancer Cells

**Figure 3:**
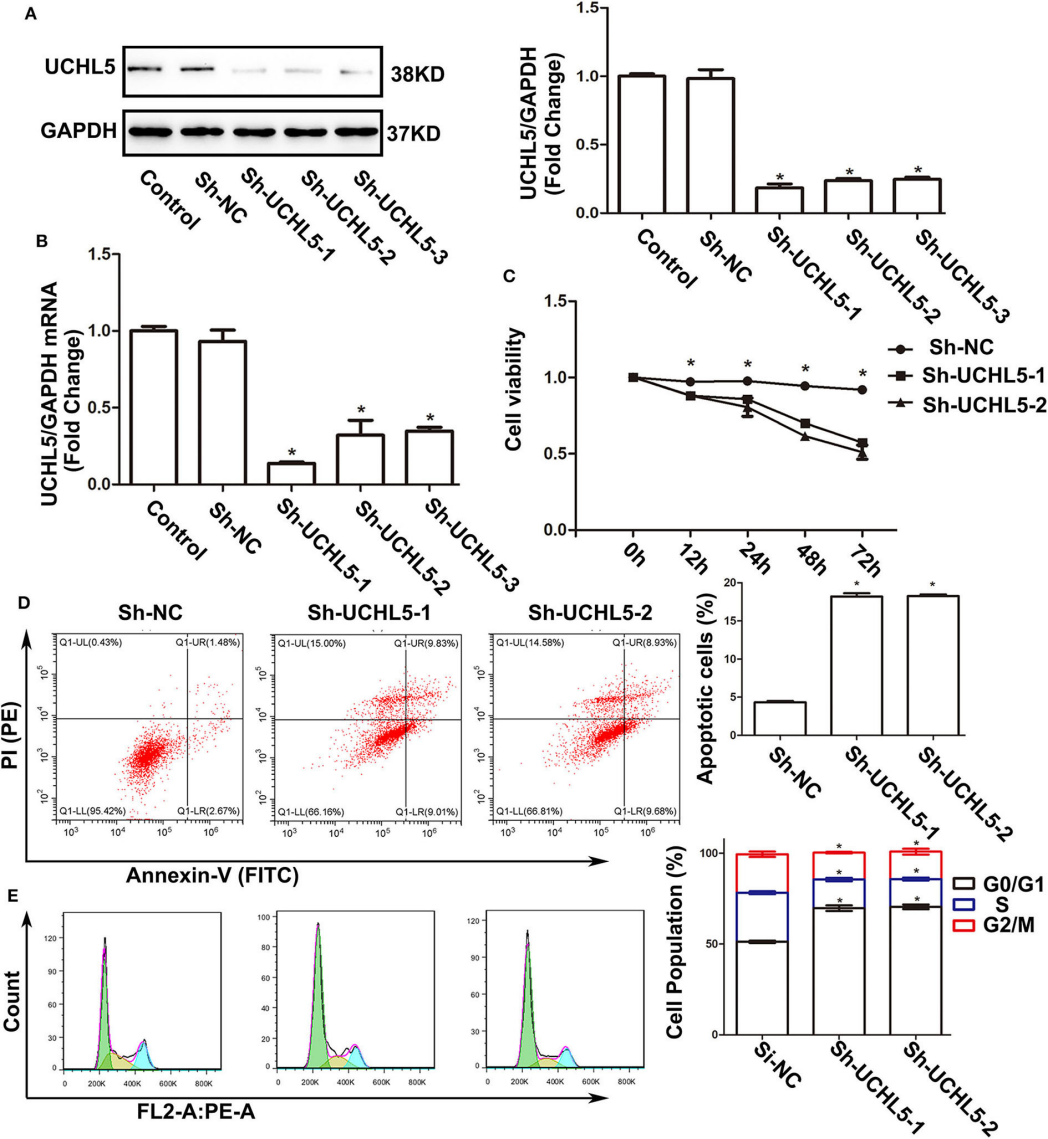
UCHL5 knockdown decreased cell viability, increased apoptosis, and arrested cell cycle in HCE-1-A endometrial cancer cells. **(A)** The levels of UCHL5 were detected by WB in HCE-1-A cells at 48 h after shRNA lentiviral infection (*N* = 3 times). *P < 0.05 vs. control. **(B)** Relative mRNA levels of UCHL5 in shRNA lentiviral infected HCE-1-A cells (*N* = 3). *P < 0.05 vs. control. **(C)** Relative cell viability was detected by CCK-8 at different time points (*N* = 3). *P < 0.05 vs. sh-UCHL5-1 and sh-UCHL5-2. **(D)** Apoptosis cells were marked by Annexin V-FITC/PI, and the percentage was analyzed by flow cytometry in HCE-1-A cells at 48 h after shRNA lentiviral infection (*N* ≥ 3). *P < 0.05 vs. sh-NC (negative control). **(E)** Cell cycle was measured by Cell Cycle Analysis Kit (*N* ≥ 3). *P < 0.05 vs. sh-NC.

Since the expression of UCHL5 was highest in HCE-1-A endometrial cancer cells, we knocked down UCHL5 expression using lentiviral-mediated shRNA in HCE-1-A cells to demonstrate the actions of UCHL5 on tumorigenesis. All three shRNA sequences significantly reduced the expression of UCHL5 in protein and mRNA levels in HCE-1-A cells (Figures 3A,B). UCHL5 knockdown descended the relative viability of HCE-1-A cells measured by CCK-8 kits (Figure 3C) and augmented the percentage of apoptosis cells (Figure 3D). Furthermore, we performed flow cytometry to examine the cell cycle distribution after treatment. As shown in Figure 3E, UCHL5 silent cells showed a remarkable increase in the sub-G1 population compared to negative control, indicating that UCHL5 deficiency induced cell cycle arrest.

### Upregulation of UCHL5 Promoted the Growth of Endometrial Cancer Cells

**Figure 4:**
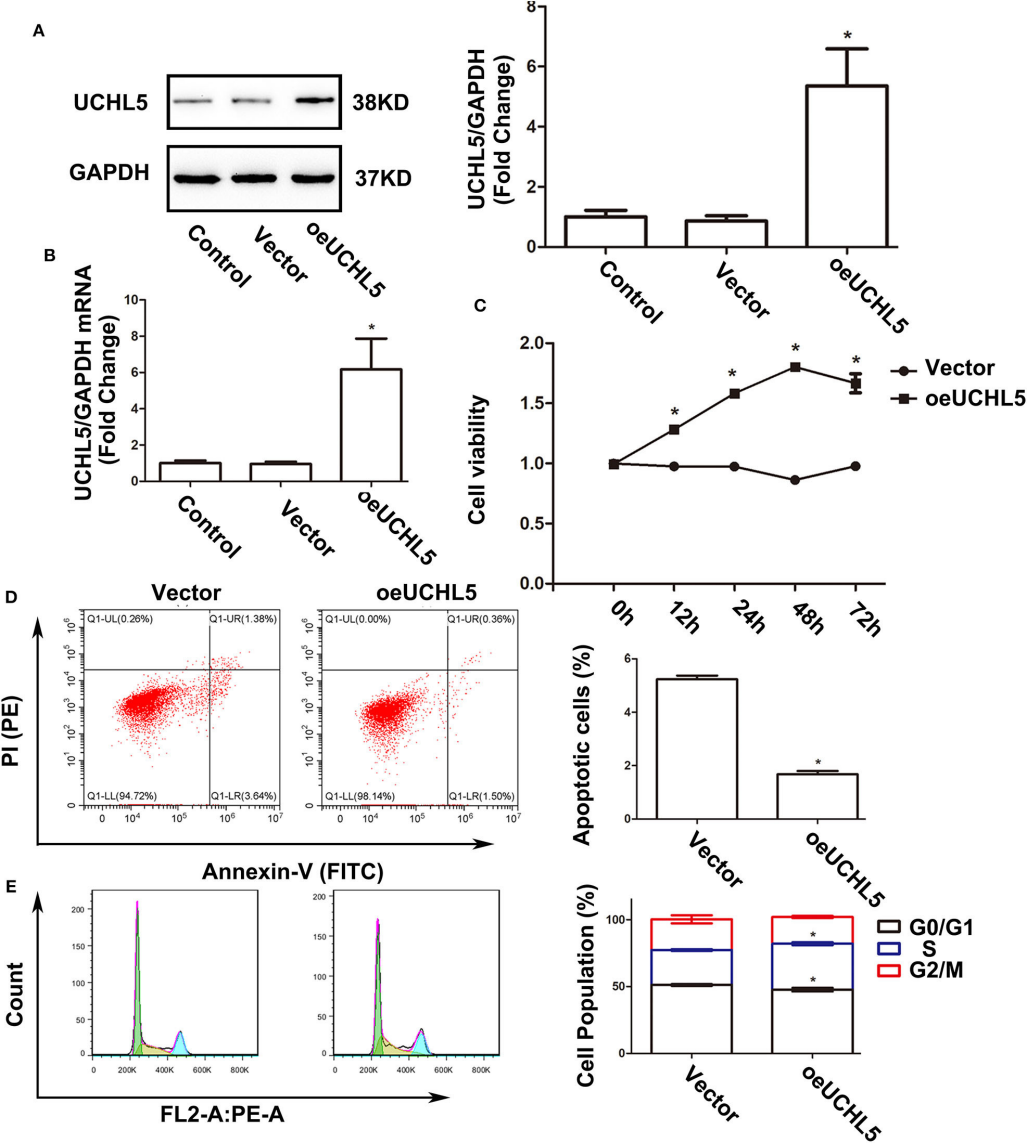
Upregulation of UCHL5 promoted cell viability, attenuated apoptosis, and accelerated cell cycle in AN3-CA cells. **(A)** The levels of UCHL5 were detected by WB in AN3-CA cells at 48 h after being infected with overexpressed lentiviral vectors (*N* = 3 times). *P < 0.05 vs. control. **(B)** Relative mRNA levels of UCHL5 measured by qRT-PCR in overexpressed lentiviral infected AN3-CA cells (*N* = 3). *P < 0.05 vs. control. **(C)** Relative cell viability was detected by CCK-8 at different time points (*N* = 3). *P < 0.05 vs. oeUCHL5 group. **(D)** Apoptosis cells were marked by Annexin V-FITC/PI, and the percentage was analyzed by flow cytometry (*N* ≥ 3). *P < 0.05 vs. empty vector. **(E)** Cell cycle was measured by Cell Cycle Analysis Kit (*N* ≥ 3). *P < 0.05 vs. empty vector.

Since the expression of UCHL5 was relatively lower in AN3-CA endometrial cancer cells, we increased the expression of UCHL5 by lentiviral infection in AN3-CA cells. By qRT-PCR and WB analysis, we demonstrated that UCHL5 gene delivery was very efficient (Figures 4A,B). UCHL5 overexpression raised cell viability at different time points after lentiviral infection (Figure 4C). Moreover, UCHL5 overexpression decreased the percentage of apoptosis cells and sub-G1 population (Figures 4D,E). These results indicated that upregulation of UCHL5 expression contributed to the growth of endometrial cancer cells.

### UCHL5 Activated Wnt/β-Catenin Signaling and Affected the Expression of Its Target Genes

**Figure 5:**
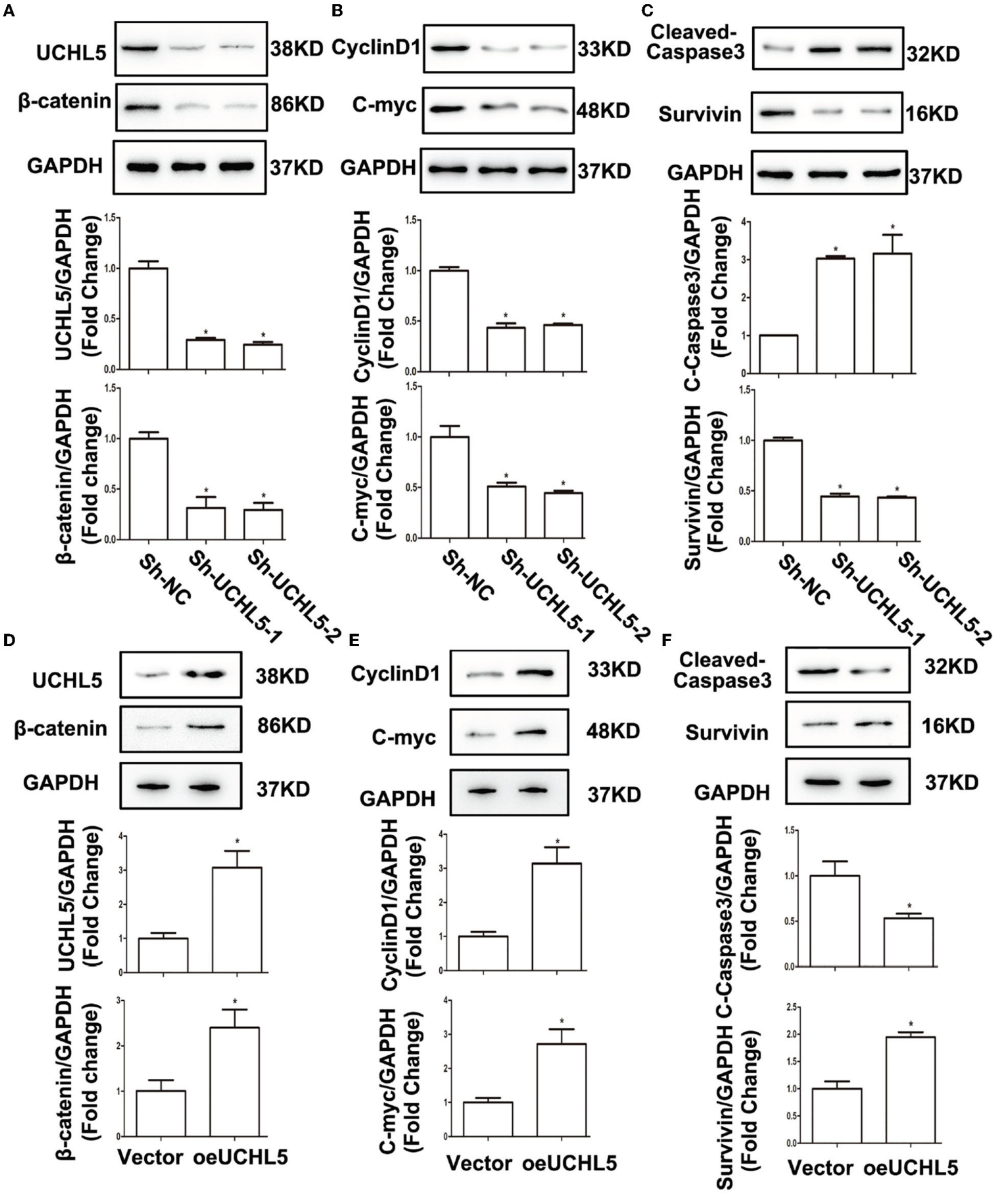
UCHL5 activated Wnt/β-catenin signaling and affected the expression of its target genes. **(A)** Relative protein levels of UCHL5 and β-catenin were detected by WB in HCE-1-A cells at 48 h after shRNA lentiviral infection (*N* = 3 times). *P < 0.05 vs. sh-NC. **(B)** Relative levels of cell cycle–related protein CyclinD1 and cell proliferation-related protein C-myc. (*N* = 3 times). *P < 0.05 vs. sh-NC. **(C)** Relative levels of cell apoptosis-related protein cleaved-caspase3 and anti-apoptosis protein Survivin (*N* = 3 times). *P < 0.05 vs. sh-NC. **(D)** Relative protein levels of UCHL5 and β-catenin were detected by WB in AN3-CA cells at 48 h after infected with overexpressed lentiviral vectors (*N* = 3 times). *P < 0.05 vs. empty vector. **(E)** Relative levels of CyclinD1 and C-myc (*N* = 3 times). *P < 0.05 vs. empty vector. **(F)** Relative levels of cleaved-caspase3 and Survivin (*N* = 3 times). *P < 0.05 vs. empty vector.

Retrospecting the bioinformatics results that UCHL5 high expression positively correlated with Wnt/β-catenin signaling, we used western blot to detect the influence of UCHL5 on β-catenin expression, a core element of the Wnt/β-catenin signaling pathway. As shown in Figure 5A, UCHL5 knockdown decreased the expression of β-catenin. CyclinD1 and C-myc and anti-apoptosis protein Survivin were significantly suppressed, but apoptosis-related protein cleaved-caspase3 was increased in the UCHL5 knockdown HCE-1-A cells compared with negative controls (Figures 5B,C). In contrast, UCHL5 overexpression in AN3-CA cells elevated the expression of β-catenin and its effector proteins (CyclinD1, C-myc, Survivin) and decreased cleaved-caspase3 (Figures 5D–F). Taking all these into consideration, UCHL5 possibly regulated cell cyclin, proliferation, and apoptosis via the Wnt/β-catenin signaling pathway in EC cells.

### Wnt/β-Catenin Signaling Pathway Inhibitor XAV939 Eliminated the Tumorigenic Effects Aroused by UCHL5 Overexpression in EC Cells

**Figure 6:**
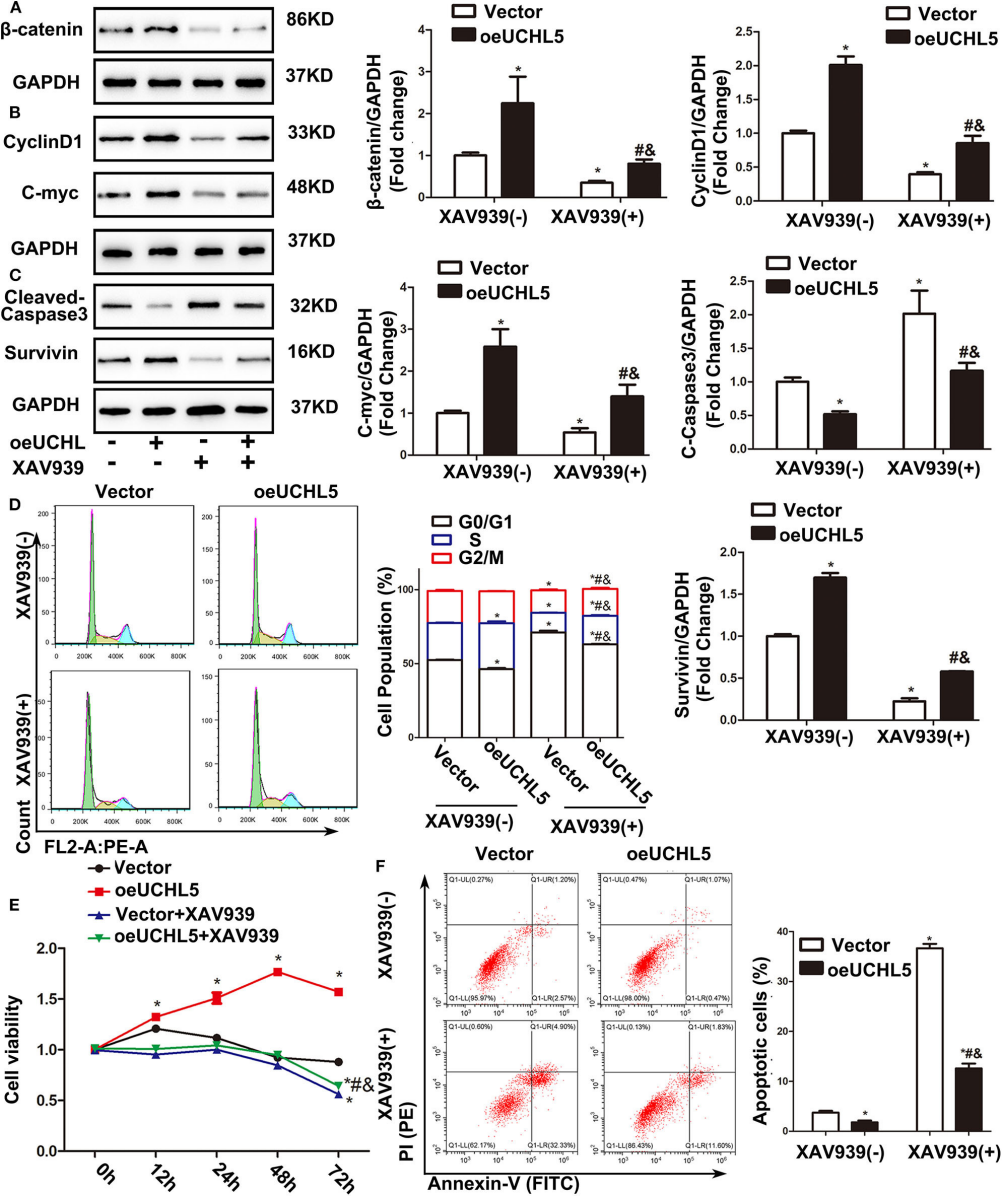
Wnt/β-catenin signaling pathway inhibitor XAV939 eliminated the disadvantages aroused by UCHL5 overexpression in AN3-CA cells. **(A)** UCHL5 overexpression (oeUCHL5). AN3-CA cells were treated with or without Wnt/β-catenin signaling pathway inhibitor XAV939 (10 μM) for 24 h. Relative protein levels of β-catenin were detected by WB. *P < 0.05 vs. empty vector; ^#^*P* < 0.05 vs. oeUCHL5; ^&^*P* < 0.05 vs. vector + XAV939. **(B,C)** Relative protein levels of CyclinD1, C-myc, cleaved-caspase3, and Survivin were detected by WB. *P < 0.05 vs. empty vector; ^#^*P* < 0.05 vs. oeUCHL5; ^&^*P* < 0.05 vs. vector + XAV939. **(D)** Cell cycle was measured by Cell Cycle Analysis Kit (*N* ≥ 3). *P < 0.05 vs. empty vector; ^#^*P* < 0.05 vs. oeUCHL5; ^&^*P* < 0.05 vs. vector + XAV939. **(E)** Relative cell viability were detected by CCK-8 at different time points (*N* = 3). *P < 0.05 vs. empty vector; ^#^*P* < 0.05 vs. oeUCHL5; ^&^*P* < 0.05 vs. vector + XAV939. **(F)** The percentage of Apoptosis cells (*N* ≥ 3). *P < 0.05 vs. empty vector; ^#^*P* < 0.05 vs. oeUCHL5; ^&^*P* < 0.05 vs. vector + XAV939.

To further confirm whether the UCHL5-regulated cell phenotype in EC cells was mediated by the Wnt/β-catenin signaling pathway, Wnt/β-catenin inhibitor XAV939 was used in the present study. As expected, UCHL5 overexpression induced the increase in β-catenin and its target genes CyclinD1, C-myc, and Survivin and the decrease in cleaved-caspase3 in AN3-CA cells, which were reversed by excessive XAV939 treatment (Figures 6A–C). Additionally, XAV939 treatment showed a smaller sub-G1 population when compared with the untreated group and prevented the cell cycle acceleration caused by UCHL5 overexpression (Figure 6D). Similar trends were observed in cell viability and apoptosis (Figures 6E,F). That is, the Wnt/β-catenin signaling pathway plays an important intermediate link between UCHL5 expression and the growth of EC cells.

### UCHL5 Silence Restricted Tumor Growth via Inhibiting the Wnt/β-Catenin Signaling Pathway *in vivo*

**Figure 7:**
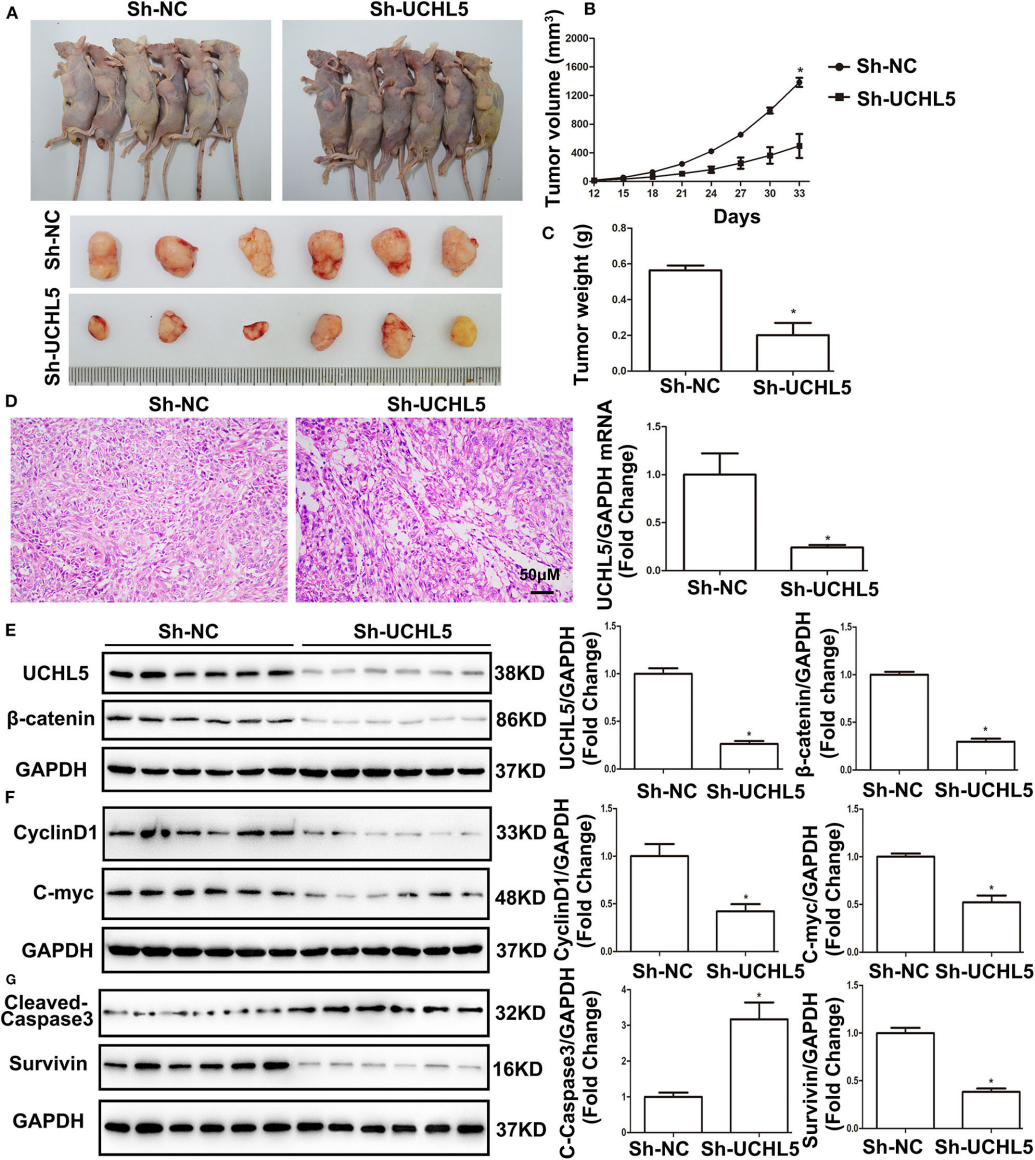
UCHL5 silence restricted tumor growth via inhibiting Wnt/β-catenin signaling pathway *in vivo*. **(A)** Images of HCE-1-A tumor-bearing nude mice and isolated primary xenograft tumors (*n* = 6). **(B)** The growth curves of HCE-1-A primary xenograft tumors (*n* = 6). *P < 0.05 vs. sh-NC. **(C)** The weight of xenograft tumors (*n* = 6). *P < 0.05 vs. sh-NC. **(D)** Hematoxylin and eosin (HE) staining of tumors (*n* = 6). **(E)** Western blot detected the expression of UCHL5 and β-catenin (*N* = 3 times). *P < 0.05 vs. sh-NC. **(F,G)** Representative bands of CyclinD1, C-myc, cleaved-caspase3, Survivin, and GAPDH and their cartogram evaluated by western blot (*N* = 3 times). *P < 0.05 vs. sh-NC.

To investigate the role of UCHL5 in tumorigenesis *in vivo*, the HCE-1-A cells stably transfected with lentivirus particles containing UCHL5 shRNA were injected subcutaneously into female nude mice. As shown in Figures 7A–C, UCHL5 silence obviously repressed the size, volume, and weight of the xenograft tumors in the nude mice. Hematoxylin–eosin (HE) staining of xenograft tumors revealed that UCHL5 knockdown presented better differentiated morphology and keratinization than the control group (Figure 7D). Consistent with the results aforementioned *in vitro*, UCHL5 silence resulted in decreases in β-catenin and its target genes CyclinD1, C-myc, and Survivin but an increase in cleaved-caspase3 *in vivo* (Figures 7E–G).

In conclusion, our results that UCHL5 promoted the growth of EC *in vivo* and *vitro* via activating the Wnt/β-catenin signaling pathway may provide potential targets for EC control in the future.

## Discussion

Considering the poor responses and prognosis of current chemotherapy drugs for EC, our enthusiasm to explore more potential anti-cancer medicine was blooming. In this study, the increase in UCHL5 expression was observed in EC tissues and negatively correlated with overall patient survival. In addition, UCHL5 overexpression mediated by lentivirus vectors activated Wnt/β-catenin signaling in endometrial cancer cells and resulted in a decrease in apoptosis and an increase in proliferation. β-Catenin upregulation was the central event because of its capacity to control the expression of cell cycle checker CyclinD1, proliferation-related C-myc, apoptosis-related cleaved-caspase 3, and Survivin.

The above results were confirmed by the fact that Wnt/β-catenin signaling inhibitor XAV939 eliminated the effects caused by UCHL5 overexpression. Additionally, UCHL5 silence mediated by sh-RNA lentivirus produced effective inhibition of EC growth *in vitro* and *vivo*. As expected, UCHL5 silence arrested cell proliferation and cycle and induced cell apoptosis via inhibiting the Wnt/β-catenin signaling pathway.

Cancer cells always display high rates of protein synthesis, and the growth of tumors depends on the ubiquitin–proteasome system (UPS) for maintenance of homeostasis (10). Whatever destroyed the UPS, cancer cells would go to die for a continuous state of proteotoxic stress caused by defective protein accumulation (16, 25). The success of proteasome inhibitors used to treat cancer in the clinic arouses great attention on ubiquitination modulators for cancer treatment (26). DUBs prevent protein degradation by removing ubiquitin chains from protein substrates. Interestingly, increasingly more and more studies found that DUBs are overexpressed and produce promoting effects in many cancer cells (25). Moreover, a series of small molecules against DUBs have been developed and implicated in cancer treatment (27). For example, USP7 inhibitor FT671 significantly inhibited the growth of medulloblastoma, colorectal, and lung tumors in mice. Moreover, b-AP15, PtPT, and VLX1570, as inhibitors of ubiquitin-specific protease 14 (USP14) and UCHL5, induce apoptosis in myeloma, breast cancer, and prostate cancer cells (28, 29). Thus, DUBs seem to be excellent drug targets for cancer treatment.

UCHL5 reversibly associates with the 26S proteasome and prevents target proteins degradation by hydrolyzing ubiquitin chains (30). Several studies have reported that high UCHL5 expression detected by immunohistochemistry or WB has a positive correlation with poor survival and high probability of recurrence (15, 31, 32). However, few researches answer whether UCHL5 is expressed on human EC tissues and if UCHL5 affects their growth and long-term outcome. This study firstly reported that UCHL5 was more highly expressed on EC tissues and cell lines and UCHL5 overexpression accelerated EC growth. These results indicated that UCHL5 was an attractive target for drug development of EC treatment. It is worth emphasizing that the current inhibitors, such as b-AP15, PtPT, and VLX1570, targeting both UCHL5 and Usp-14, showed limited application for their multiple side effects. Thus, highly selective drugs targeting UCHL5 alone deserve being explored.

β-Catenin is a key signal transduction protein in the Wnt/β-catenin pathway, which controls transcription of a wide range of genes involved in embryonic development, cell proliferation and migration, and cell fate (33). Aberrant activation of the Wnt/β-catenin pathway has been reported in EC and associates with the deterioration outcome (34, 35). Previous researches documented that deletion of UCHL5 increased the level of the ubiquitinated β-catenin and accelerated the hydrogen peroxide–stimulated degradation of β-catenin in HeLa cells (36). In the study, UCHL5 overexpression elevated the levels of β-catenin and regulated its downstream genes (CyclinD1, C-myc, Survivin, and cleaved-caspase3), which were counteracted by the Wnt/β-catenin inhibitor XAV939. The deeper mechanism may be that the degradation of β-catenin depends on the UCHL5-mediated ubiquitin–proteasome pathway. Above all, Wnt/β-catenin pathway activation at least partly explained the tumor-promoting effects of UCHL5.

Although this study seemed to draw a whole theory for the application of UCHL5 inhibition on EC treatment, the following limitations remain to be considered. Firstly, how was UCHL5 upregulated in cancer tissues? Three potential methylation sites in the UCHL5 promoter have been predicted in previous studies (37). However, more methylation sites and microRNAs may be important breakthroughs. The deubiquitination activity of UCHL5 depends on the recruitment by hRpn13 which is a component of the 19S particle and bind to UCHL5 via KEKE motifs. Thus, the levels of hRpn13 and its regulating effects on UCHL5 in EC should be detected in future studies. Additionally, previous research believed that nuclear or cytoplasmic UCHL5 expression, respectively, was reported to be a predictor for survival in several cancers (38, 39). However, the difference of the expression and function between cytoplasmic and nucleus UCHL5 was not compared in current known studies.

In summary, this study provides new insights into the effects of the deubiquitinating enzyme UCHL5 in EC. UCHL5 not only overexpressed on EC tissues and cell lines but also promoted their growth. We suspect that it is because UCHL5 rescues the ubiquitination degradation of β-catenin. The elevated β-catenin stimulated cell proliferation, accelerated cell cycle, and decreased apoptosis via regulating its downstream proteins in EC cells. Thus, UCHL5 plays an important role in EC growth and is hopefully developed into a new therapeutic strategy for EC.

## Data Availability Statement

The datasets generated for this study are available on request to the corresponding author.

## Ethics Statement

The studies involving human participants were reviewed and approved by Ethics Committee of Shengjing Hospital affiliated with China Medical University. The patients/participants provided their written informed consent to participate in this study. The animal study was reviewed and approved by Ethics Committee of Shengjing Hospital affiliated with China Medical University.

## Author Contributions

DL and LO developed the hypothesis, designed the experiments, and wrote this manuscript. DL, ZS, and XW performed the experiments and analyzed the data.

## Conflict of Interest

The authors declare that the research was conducted in the absence of any commercial or financial relationships that could be construed as a potential conflict of interest.
