# Corrigendum: Ubiquitin C-Terminal hydrolase L5 (UCHL5) accelerates the growth of endometrial cancer *via* activating the Wnt/β-catenin signaling pathway

**DOI:** 10.3389/fonc.2022.992496

**Published:** 2022-08-17

**Authors:** Da Liu, Zixuan Song, Xiaoying Wang, Ling Ouyang

**Affiliations:** Department of Obstetrics and Gynecology, Shengjing Hospital of China Medical University, Shenyang, China

**Keywords:** endometrial cancer, UCHL5, lentivirus vectors, Wnt/β-catenin pathway, XAV939

In the published article, there was an operator error in the western blot images of **Figure 5**. The original images provided by the authors were not spliced. The corrected **Figure 5** and its caption “UCHL5 activated Wnt/β-catenin signaling and affected the expression of its target genes. (A) Relative protein levels of UCHL5 and β-catenin were detected by WB in HCE-1-A cells at 48 h after shRNA lentiviral infection (*N* = 3 times). *P < 0.05 vs. sh-NC. (B) Relative levels of cell cycle–related protein CyclinD1 and cell proliferation-related protein C-myc. (*N* = 3 times). *P < 0.05 vs. sh-NC. (C) Relative levels of cell apoptosis-related protein cleaved-caspase3 and anti-apoptosis protein Survivin (*N* = 3 times). *P < 0.05 vs. sh-NC. (D) Relative protein levels of UCHL5 and β-catenin were detected by WB in AN3-CA cells at 48 h after infected with overexpressed lentiviral vectors (*N* = 3 times). *P < 0.05 vs. empty vector. (E) Relative levels of CyclinD1 and C-myc (*N* = 3 times). *P < 0.05 vs. empty vector. (F) Relative levels of cleaved-caspase3 and Survivin (*N* = 3 times). *P < 0.05 vs. empty vector.” appear below.

**Figure 5:**
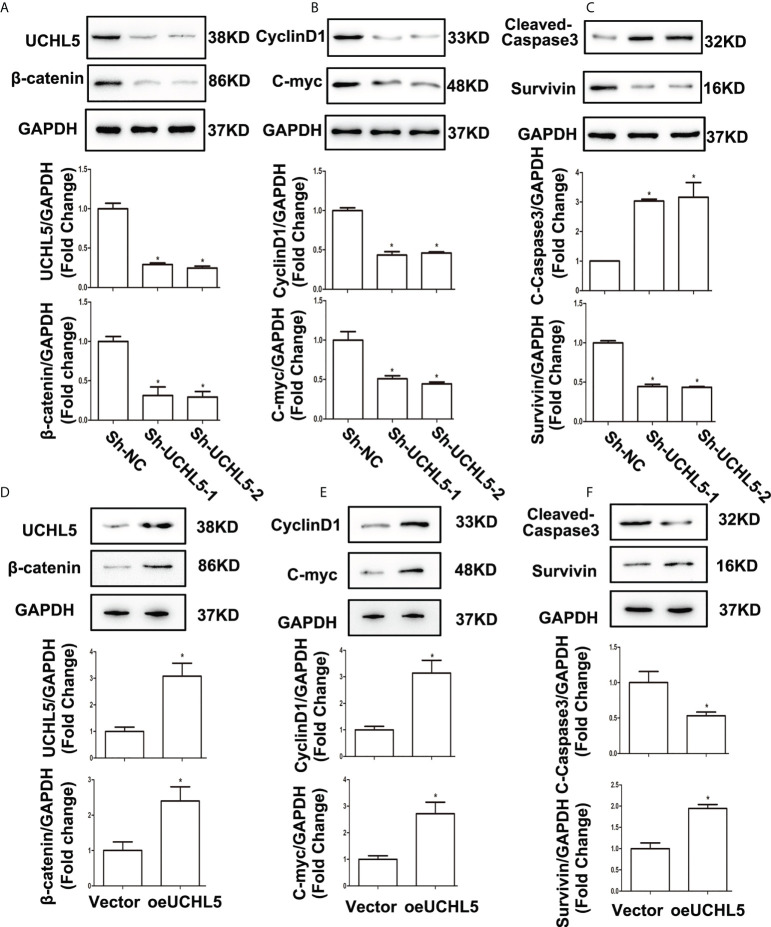
UCHL5 activated Wnt/β-catenin signaling and affected the expression of its target genes. **(A)** Relative protein levels of UCHL5 and β-catenin were detected by WB in HCE-1-A cells at 48 h after shRNA lentiviral infection (*N* = 3 times). *P < 0.05 vs. sh-NC. **(B)** Relative levels of cell cycle–related protein CyclinD1 and cell proliferation-related protein C-myc. (*N* = 3 times). *P < 0.05 vs. sh-NC. **(C)** Relative levels of cell apoptosis-related protein cleaved-caspase3 and anti-apoptosis protein Survivin (*N* = 3 times). *P < 0.05 vs. sh-NC. **(D)** Relative protein levels of UCHL5 and β-catenin were detected by WB in AN3-CA cells at 48 h after infected with overexpressed lentiviral vectors (*N* = 3 times). *P < 0.05 vs. empty vector. **(E)** Relative levels of CyclinD1 and C-myc (*N* = 3 times). *P < 0.05 vs. empty vector. **(F)** Relative levels of cleavedcaspase3 and Survivin (*N* = 3 times). *P < 0.05 vs. empty vector.

The authors apologize for this error and state that this does not change the scientific conclusions of the article in any way. The original article has been updated.

## Publisher’s note

All claims expressed in this article are solely those of the authors and do not necessarily represent those of their affiliated organizations, or those of the publisher, the editors and the reviewers. Any product that may be evaluated in this article, or claim that may be made by its manufacturer, is not guaranteed or endorsed by the publisher.

